# The Miller Hospital, Greenwich

**Published:** 1902-05-10

**Authors:** 


### THE MILLER HOSPITAL, GREENWICH.

### ANNUAL MEETING.

The annual meeting of the Miller Hospital and Royal Kent Dispensary was held on Tuesday evening, April 29th, and although about 1,000 notices had been sent out to the governors, only some seventeen of the committee attended, this being about 21 per cent. of the number serving. A spirit of unanimity and cordiality pervaded the meeting, which was presided over for the first time by the Mayor of Greenwich, Ion Hamilton Benn, Esq., J.P., who has recently taken up the cause of the hospital, and from whose interest great things are anticipated.

In moving the adoption of the report, the chairman said the record of the past year's work showed that in every respect there was improvement; numbers, work, and money all showed an increase. Subscriptions seemed to have fairly balanced the outgoings.

#### Local Support.

He believed there were in the neighbourhood numbers of generous people, and that, although the metropolitan hospitals received a large amount of support annually from Greenwich and the neighbouring boroughs, yet if the work of the Miller Hospital were brought home to the people of Blackheath, Charlton, Kidbrook, etc., additional help might be obtained. He would suggest to the committee that some way might be found of bringing the needs of the hospital before them more fully than had been done in the past. He himself, until he came down to Greenwich and joined the borough council, had no knowledge of the hospital, otherwise, for many years past, a subscription from him might have been obtained. It was a hospital to be proud of, and he would urge that local hospitals had a claim on local people.

EXTENSION.

He hoped that next year's report would contain some particulars about the satisfactory progress in the building of the new wing. It was known to them all that the money collected at the Coronation festivities in Greenwich would be devoted to the Fund, and he was able to say that £1,750 had been already promised in addition to the £250 which the hospital had received direct from an anonymous donor.

PAYMENT BY PATIENTS.

The Mayor further said that a friend of his own had offered to give a substantial sum to the Building Fund if some system of small payments by out-patients were arranged—a system analogous to that in use at the London, Guy's, Poplar, and some other hospitals. He was not proposing to bring the matter formally before the committee, but merely to let them know that substantial assistance could be obtained, and to ask them to consider whether the plan could be adopted or not. Personally, he thought every encouragement should be given to the deserving poor, and he believed they themselves liked to pay something for the help they received. Of course the threepences at the London Hospital represented only a very small fraction of the benefit; still, they amounted to something like £1,500 a year. It had been suggested that King Edward's Fund and the other funds might reduce the amount of their grant if the system were introduced, but he believed those funds gave largely where the rule existed, and if the extension were built, he hoped they might look for a larger grant instead of a smaller one.

Mr. Councillor Sydney said the committee were very desirous to carry out the extension. The question raised by the chairman was a very difficult one, and he could promise that it should have their serious attention; but he understood King Edward's Fund made it a *sine qua non* that no charge should be made, and they had even to pay for the washing of patients' linen.

The Mayor said he appreciated the difficulty; but perhaps the committee might interview the council of King Edward's Fund, and find out their views.

A committee member remarked that "workpeople's contributions" amounted to £45 18s.; these were quite voluntary payments.

The senior surgeon, Dr. Charles H. Hartt, who, it was remarked, was one of the most regular of committee members in attendance at meetings, said the extension was greatly needed; the arrangements were by no means adequate to the growing requirements of the hospital. When he first came, there was no hospital nearer than Guy's, and broken limbs and typhoid cases had to bear the jolting over rough roads in order to obtain treatment. The work had grown enormously, and it had now become absolutely necessary to enlarge the Miller Hospital; the additional expense need not be very great in regard to staff; the two house surgeons could manage a hospital twice the size. The question of payment had come before the committee twenty years ago, and at that time it was not thought desirable to introduce it.

THE X-RAY DEPARTMENT.

A very cordial vote of thanks was passed to Mr. J. J. Vezey, F.R.M.S., for his valuable work in the X-ray department, which had been enlarged as a memorial of the late Mr. Thomas More, senior surgeon, who founded it in 1896. It was well provided with instruments, including a high-frequency apparatus which was much in request. Demonstrations to subscribers and friends were given in October, and these were largely attended. The average monthly attendances of patients numbered 58. By arrangement with medical men, private cases were treated, and the fees received covered all expenses in this branch. Although a layman, Mr. Vezey worked in entire harmony with the medical staff.

A special vote of thanks to the Press closed the proceedings.

